# Preschool diets in children from Piła, Poland, require urgent intervention as implied by high risk of nutrient inadequacies

**DOI:** 10.1186/s41043-016-0050-4

**Published:** 2016-04-19

**Authors:** Sylwia Merkiel, Wojciech Chalcarz

**Affiliations:** Food and Nutrition Department, The Eugeniusz Piasecki University School of Physical Education in Poznan, Królowej Jadwigi 27/39 Street, 61-871 Poznan, Poland

**Keywords:** Preschool children, Preschools, Dietary intake, Macronutrients, Vitamins, Minerals, Diet-related diseases

## Abstract

**Background:**

Among the studies published after the year 2000 which focused on nutrition at preschool, only three aimed to assess children’s intake of energy and selected nutrients at preschool. The purpose of this study was to assess dietary intake in children during their stay at preschool.

**Methods:**

The studied population comprised 128 4–6-year-old children who attended preschools in Piła, Poland. Intakes of energy and macronutrients were estimated from a 5-day weighed food record completed by the preschool staff. Weight and height were measured, and BMI was calculated. Statistical analysis was carried out using the IBM SPSS Statistics 21.0 computer programme. The data were analysed according to gender.

**Results:**

Energy intake was the lowest in children with underweight, 2004 kJ (478 kcal), and the highest in obese children, 3388 kJ (809 kcal). Energy intake from lactose was statistically significantly higher in boys than in girls, 3.0 vs 2.6 %. Statistically significantly higher percentage of boys in comparison to girls had intakes of vitamin C below 70 % of EAR, 56.9 vs 38.1 %. It is important to highlight the excessive intake of energy from saturated fatty acids and energy from sucrose, along with inadequate intake of energy from polyunsaturated fatty acids. We also found excessive intake of sodium and inadequate intakes of dietary fibre, water, vitamin D, vitamin E, folate, niacin, calcium and potassium.

**Conclusions:**

Preschool diets need urgent improvement to prevent diet-related diseases in the studied preschoolers in the future. The inadequacies observed in these diets are in accordance with the previously reported inadequacies in menus planned for preschoolers. More research is needed to investigate dietary intake of children during their stay at preschool. Common regulations worked out for all preschools in the European Union would be a good way to provide adequate nutrition to preschool children.

## Background

Nutrition in preschool age plays an important role as a significant factor which influences growth and development, as well as future risk of diet-related diseases [1, 2]. In Poland, as many as 69.9 % of children aged 3 to 6 years attend preschools and this percentage has been increasing since 2005 [3]. Children spend at preschools most of the time: 8 h a day and 5 days a week, and that is why preschool diet has considerable contribution to preschoolers’ daily dietary intake. Therefore, it is of greatest importance to provide balanced meals for children at preschools.

Most of the studies published after the year 2000 which focused on nutrition at preschool aimed to analyse and assess preschool menus [4–25] or to compare menus to actual foods and beverages served to children [26, 27]. In one study [28], foods offered to children were assessed using the Healthy Eating Index 2005. Two studies [29, 30] reported children’s intake of selected foods at preschool. Only three studies, carried out in Poland [31], the USA [32] and Sweden [33], aimed to assess children’s intake of energy and selected nutrients at preschools. In the study carried out in Szczecin, Poland [31], on a group of 78 children aged 4 to 6 years, intakes of energy and 20 nutrients at preschool were assessed using the method of a 3-day estimated food record. The authors also compared these intakes to the preschool menu and they reported children’s dietary intake at home. One of the purposes of the study carried out in New York City, USA [32], was to assess dietary intake in 240 preschool children aged 3 to 4 years. The authors used the method of direct observation in order to record children’s food intakes and they reported energy intake, as well as intakes of two macronutrients, 10 vitamins and five minerals. The study conducted in a suburb of Stockholm, Sweden [33], in a group of 109 preschool children aged 3 to 5 years included intakes of energy and 12 nutrients at preschool. In this study, weighed food record at preschool was used and intakes of energy, five macronutrients, three vitamins and four minerals were reported. It is important to note that all of the abovementioned studies which aimed to assess children’s food intake at preschool, except for two studies [31, 33], were carried out in the USA [26–30, 32, 33].

Thus, assessing dietary intake of children at preschools is of great relevance. Future studies should fill this gap, and dietary intake at preschools in children from the European countries should be investigated. It is particularly important in the case of the countries which are European Union members because common regulations concerning nutrition at preschools may be imposed.

Therefore, the aim of this study was to assess dietary intake in Polish children during their stay at preschool.

## Methods

### Subjects

We randomly selected two preschools in Piła, a city located in north-western part of Poland. The directors of the preschools agreed to participate in the study. Parents of 234 children, that is all children who attended these preschools, were invited to take part in the study. Written consent was provided by parents of 154 children. However, at the very beginning of the study, parents withdrew 19 children either because their child fell ill or without giving any reason. The data obtained for two girls had to be excluded from the analysis because the girls had problems with adapting themselves to the new environment and their reaction to this stressful situation was refusal to eat almost all of the foodstuffs and dishes served at preschool. The children were aged 4 to 6 years, except for five 3-year-olds who were excluded from the analysis because they fall in a different age category in the dietary reference values. Therefore, the final population comprised 128 children, 63 girls and 65 boys, aged 4 to 6 years. The study was approved by the Bioethics Committee of the Poznan University of Medical Sciences.

### Dietary intake

#### Data collection

Dietary intake was estimated from a 5-day weighed food record completed by the preschool staff. The food record covered only the time when the children stayed at preschool. The preschool staff was provided with scales to weigh all the foods and beverages served to each child. The members of the staff who were responsible for weighing were instructed how to do this and how to fill in the food diaries. The kitchen staff provided detailed information about the way of preparing meals, that is recipes, ingredients, cooking methods, etc. The preschool staff were also asked to weigh and write down all the foods and beverages which the children brought from home and ate at preschool. At the end of the data collection, 16 children fell ill. As a result, we obtained data from 5 days in the case of 112 children, data from 4 days in the case of 11 children and data from 3 days in the case of 5 children.

#### Dietary assessment

Dietary intake was calculated using the Dieta computer programme, version 4.0, worked out by the National Food and Nutrition Institute in Warsaw, Poland. The Dieta contains food composition database based on Polish food composition tables [34]. The programme was described in details in the previous articles [35, 36]. The database of the programme includes also nutritional value of typical Polish dishes; however, we did not use it. This is because the recipes applied by the National Food and Nutrition Institute in Warsaw in the Polish food composition tables [34] were different from the recipes used by the preschool kitchen staff. Therefore, we calculated nutritional value of the dishes served at preschools based on the recipes provided by the kitchen staff including the losses of nutrients resulting from food processing.

We obtained from the Dieta total energy intake (kJ, kcal) and intakes of 37 nutrients. Additionally, we used the Microsoft Excel 2010 to calculate total energy and total protein intakes per kilogramme of body weight, and animal and plant protein intakes expressed as percent of total protein intake. Although the Dieta provides the calculations of available carbohydrates, as well as energy from total protein, total fat and available carbohydrates, we had to calculate these in the Excel since we added nutritional values of some foodstuffs and dishes, as described in the previous paragraph. We calculated available carbohydrate intake as the difference between total carbohydrates and dietary fibre, that is in the same way as available carbohydrate intake obtained from the Dieta. Additionally, we calculated energy from fatty acids, lactose, sucrose and starch using the Excel. As mentioned in the previous article [35], total carbohydrate intake calculated by the Dieta based on Polish food composition tables was derived ‘by difference’, while dietary fibre intake calculated by the Dieta means dietary fibre determined using enzymatic-gravimetric method (AOAC 1990) [34, 37]. Total water intake obtained from the Dieta includes both water from beverages and water from food.

#### Comparison with nutritional guidelines

Energy intake from macronutrients and intake of cholesterol were compared to the recommendations in the prevention of diet-related diseases [38] similarly to the previous article [35]. To assess nutrient intakes, dietary reference values for Polish population [39] were used: Estimated Average Requirement (EAR) in the case of total protein (g/kg), vitamin A, B_1_, B_2_, B_6_, folate, vitamin B_12_, niacin, vitamin C, calcium, phosphorus, magnesium, iron, zinc, copper and iodine, and Adequate Intake (AI) in the case of dietary fibre, total water, vitamin E, sodium and potassium. Intake of vitamin D was compared to EAR worked out by the Food and Nutrition Board of the Institute of Medicine [40] because Polish dietary reference intakes include only AI. To assess manganese intake, we used AI worked out by the Food and Nutrition Board of the Institute of Medicine [41] because Polish dietary reference intakes do not include this mineral [39]. However, all intakes were compared to 70 % of EAR or 70 % of AI, because according to the Polish recommendations preschool meals should provide 70 % of dietary reference values [42].

Nutrient intakes were also compared to 70 % of Tolerable Upper Intake Level (UL) if available. Polish dietary reference values include UL only for sodium [39]. Therefore, we used UL worked out by the Scientific Committee on Food [43] in the case of retinol, vitamin D, E, B_6_, folate, zinc, copper and iodine, and UL worked out by the Food and Nutrition Board of the Institute of Medicine [40, 41, 44–46] in the case of niacin, vitamin C, calcium, phosphorus, iron and manganese. We did not compare magnesium intake to UL since the UL was established for magnesium from nonfood sources, and the studied children did not take magnesium supplements.

### Anthropometric measures

Weight and height were measured, and body mass index (BMI) was calculated, using the methods described in our previous article [47]. BMI was classified into percentile ranges using the tables provided by Kuczmarski et al. [48]. The percentile ranges were classified using the terminology recommended by the International Obesity Task Force [49]: below the 5th percentile—underweight; from the 5th to the 84th percentile—healthy weight; from the 85th to the 94th percentile—overweight; the 95th percentile or above—obesity [35, 47].

### Statistical analysis

Statistical analysis was carried out using the IBM SPSS Statistics for Windows computer programme, version 21.0 (Armonk, NY: IBM Corp.). The data were analysed according to gender, except for energy intake which was also analysed according to the percentile categories for BMI. Means, standard deviations (SD), medians and standard errors (SE) were calculated for energy and nutrient intakes. In the case of total protein (% of energy), total fat (% of energy), saturated fatty acids (% of energy), polyunsaturated fatty acids (% of energy), monounsaturated fatty acids (% of energy), cholesterol (mg) and available carbohydrates (% of energy), the percentages of children with nutrient intakes below, within or above the recommendations were calculated. To investigate the prevalence of inadequate intake, we calculated the percentages of children with intakes below 70 % of EAR. Additionally, we calculated the percentages of children whose nutrient intakes were below 70 % of AI, as in the previous studies [35, 50], although it is important to note that AI cannot be used to estimate the prevalence of inadequate nutrient intakes for groups [51]. We also calculated the percentages of children with vitamin and mineral intakes above 70 % of UL.

Statistical significance for qualitative variables was determined using Pearson’s chi-square test. In the case of quantitative variables, the Shapiro-Wilk statistic for testing normality was used. Unpaired Student’s *t* test was applied to investigate statistically significant differences for normally distributed variables and the non-parametric Mann–Whitney *U* test was used in the case of skewed variables. The differences were considered significant at *P* ≤ 0.05.

## Results

Socio-demographic characteristics of the studied preschool children were presented in the previous article [52].

Table 1 shows energy intake in the studied children according to the percentile categories for BMI. Energy intake was the lowest in children with underweight, 2004 kJ (478 kcal), and the highest in obese children, 3388 kJ (809 kcal).

**Table 1:** Energy intake in the studied preschool children according to the percentile categories for BMI

Percentile categories for BMI	Energy intake (kJ) [Energy intake (kcal)]				Population	
	Mean	SD	Median	SE	%	*n*
Below the 5th percentile (underweight)	2004	676	1758	302	3.9	5
	[478]	[161]	[419]	[72]		
5th–84th percentile (healthy weight)	2878	695	2929	72	72.6	93
	[687]	[166]	[700]	[17]		
85th–94th percentile (overweight)	3020	709	3116	148	18.0	23
	[721]	[169]	[743]	[35]		
95th percentile and above (obesity)	3388	789	2950	298	5.5	7
	[809]	[189]	[705]	[71]		

Table 2 presents energy and macronutrient intakes in the studied children, and Table 3 shows the percentages of the studied children in the reference ranges for macronutrient intake. Statistically significant difference was found only for energy intake from lactose, which was higher in boys than in girls, 3.0 % vs 2.6 %. It is important to highlight the excessive intake of energy from saturated fatty acids (mean intake of 13.9 % and as many as 93.0 % of children with intakes above the recommendations) along with inadequate intake of energy from polyunsaturated fatty acids (mean intake of 3.0 % and all children with intakes below the recommendations). Intake of energy from sucrose was very high, 20.3 %, while intakes of dietary fibre, 5.5 g, and water, 727 g, were very low.

**Table 2:** Energy and macronutrient intakes in the studied children during their stay at preschool

Energy/nutrient	Reference values	Girls (*n* = 63)		Boys (*n* = 65)		All children (*n* = 128)		*P*	Girls (*n* = 63)		Boys (*n* = 65)		All children (*n* = 128)	
		Mean	SD	Mean	SD	Mean	SD		Median	SE	Median	SE	Median	SE
Energy														
(kJ)	Body weight dependent	2922	732	2872	728	2897	727	NS	2961	92	2914	90	2939	64
(kcal)		697	175	685	174	691	174	NS	707	22	695	22	702	15
(kJ/kg body weight)	NA	145	35	142	37	143	36	NS	148	4	144	5	144	3
(kcal/kg body weight)	NA	35	8	34	9	34	9	NS	35	1	34	1	34	1
Total protein														
(g)	Body weight dependent	20.2	5.7	20.0	5.7	20.1	5.7	NS	20.8	0.7	20.3	0.7	20.7	0.5
(g/kg body weight)	0.59^a^	1.0	0.3	1.0	0.3	1.0	0.3	NS	1.0	0.0	1.0	0.0	1.0	0.0
(% of energy)	10–15 %	11.5	1.3	11.6	0.9	11.6	1.1	NS	11.5	0.2	11.7	0.1	11.6	0.1
Animal protein														
(g)	NA	11.7	3.9	11.9	4.3	11.8	4.1	NS	12.1	0.5	11.6	0.5	12.1	0.4
(% of total protein)	NA	56.4	9.6	58.4	8.4	57.4	9.0	NS	58.0	1.2	59.8	1.0	58.8	0.8
Plant protein														
(g)	NA	8.6	2.5	8.1	2.2	8.3	2.4	NS	8.4	0.3	8.0	0.3	8.2	0.2
(% of total protein)	NA	43.4	9.7	41.3	8.6	42.4	9.2	NS	42.0	1.2	39.6	1.1	41.2	0.8
Total fat														
(g)	NA	24.1	7.5	23.9	8.0	24.0	7.8	NS	24.4	0.9	24.0	1.0	24.3	0.7
(% of energy)	20–30 %	30.7	3.7	30.8	4.5	30.7	4.1	NS	31.2	0.5	32.1	0.6	31.6	0.4
Saturated fatty acids														
(g)	NA	10.81	3.72	10.95	3.88	10.88	3.79	NS	11.22	0.47	11.05	0.48	11.21	0.33
(% of energy)	<10 %	13.7	2.3	14.0	2.4	13.9	2.4	NS	13.8	0.3	14.6	0.3	14.2	0.2
Polyunsaturated fatty acids														
(g)	NA	2.37	0.65	2.26	0.66	2.31	0.65	NS	2.38	0.08	2.25	0.08	2.30	0.06
(% of energy)	6–10 %	3.1	0.4	3.0	0.4	3.0	0.4	NS	3.0	0.1	3.0	0.0	3.0	0.0
Monounsaturated fatty acids														
(g)	NA	9.26	2.85	9.03	3.07	9.14	2.96	NS	9.53	0.36	9.30	0.38	9.51	0.26
(% of energy)	>10 %^b^	11.8	1.6	11.7	1.9	11.7	1.8	NS	12.0	0.2	12.3	0.2	12.1	0.2
Cholesterol														
(mg)	<210^c^	99	31	98	38	98	34	NS	102	4	94	5	101	3
Total carbohydrates														
(g)	NA	105.0	24.6	102.3	23.0	103.6	23.7	NS	104.3	3.1	106.4	2.8	105.5	2.1
Available carbohydrates														
(g)	91^d^	99.2	23.2	97.0	21.8	98.1	22.4	NS	99.4	2.9	100.9	2.7	99.8	2.0
(% of energy)	55–70 %^e^	57.5	4.2	57.3	4.8	57.4	4.5	NS	56.7	0.5	55.9	0.6	56.4	0.4
Lactose														
(g)	NA	4.7	2.2	5.2	2.1	4.9	2.2	NS	4.9	0.3	5.0	0.3	5.0	0.2
(% of energy)	NA	2.6	1.0	3.0	1.0	2.8	1.0	0.033	2.6	0.1	2.9	0.1	2.8	0.1
Sucrose														
(g)	NA	34.1	8.0	34.6	9.0	34.3	8.5	NS	34.1	1.0	33.8	1.1	34.0	0.8
(% of energy)	NA	20.1	4.2	20.6	3.7	20.3	4.0	NS	19.8	0.5	20.5	0.5	20.2	0.4
Starch														
(g)	NA	52.2	15.5	49.6	13.9	50.9	14.7	NS	52.5	2.0	49.4	1.7	50.3	1.3
(% of energy)	NA	30.0	4.6	29.2	5.3	29.6	5.0	NS	29.5	0.6	28.7	0.7	29.1	0.4
Dietary fibre														
(g)	9.8^f^	5.7	1.6	5.3	1.4	5.5	1.5	NS	5.6	0.2	5.3	0.2	5.5	0.1
Total water														
(g)	1120^f^	742	165	713	148	727	157	NS	747	21	717	18	734	14

*P* significance, *NA* not available, *NS* not significant (*P* > 0.05)

^a^70 % EAR

^b^Calculated by difference as: total fat − (saturated fatty acids + polyunsaturated fatty acids)

^c^70 % of the WHO recommendations

^d^70 % RDA

^e^Calculated by difference: as the percentage of total energy − energy from total protein − energy from total fat

^f^70 % AI

**Table 3:** The percentages of the studied children in the reference ranges for macronutrient intake during their stay at preschool

Nutrient	Girls (*n* = 63)	Boys (*n* = 65)	All children (*n* = 128)	*P*
	%	%	%	
Total protein (% of energy)				
Below the recommendations	3.2	4.6	3.9	NS
Within the recommendations	96.8	95.4	96.1	
Total fat (% of energy)				
Below the recommendations	1.6	3.1	2.3	NS
Within the recommendations	36.5	33.8	35.2	
Above the recommendations	61.9	63.1	62.5	
Saturated fatty acids (% of energy)				
Within the recommendations	7.9	6.2	7.0	NS
Above the recommendations	92.1	93.8	93.0	
Polyunsaturated fatty acids (% of energy)				
Below the recommendations	100.0	100.0	100.0	#
Monounsaturated fatty acids (% of energy)				
Below the recommendations	15.9	24.6	20.3	NS
Within the recommendations	84.1	75.4	79.7	
Cholesterol (mg)				
Within the recommendations	100.0	100.0	100.0	#
Available carbohydrates (% of energy)				
Below the recommendations	23.8	41.5	32.8	NS
Within the recommendations	73.0	56.9	64.8	
Above the recommendations	3.2	1.5	2.3	
Dietary fibre (g)				
Below 70 % AI	100.0	100.0	100.0	#
Total water (g)				
Below 70 % AI	100.0	100.0	100.0	#

*P* significance

#*P* cannot be calculated when percentage is 0.0 or 100.0

Tables 4 and 5 show vitamin and mineral intake in the studied children, respectively, whereas Tables 6 and 7 present the percentages of the studied children in the reference ranges for vitamin and mineral intake, respectively. Statistically significantly higher percentage of boys in comparison to girls had intakes of vitamin C below 70 % of EAR, 56.9 vs 38.1 %. Intakes of vitamin D and calcium were well below 70 % of EAR, 0.59 μg and 195 mg, respectively. Also intakes of folate and niacin were lower than 70 % of EAR, 84.8 μg and 3.72 mg, respectively. Intakes of vitamin E and potassium were below 70 % of AI, 2.31 and 947 mg, respectively, whereas intake of sodium was higher than 70 % of UL in as many as 71.1 % of the studied children.

**Table 4:** Vitamin intake in the studied children during their stay at preschool

Nutrient	Reference values	Girls (*n* = 63)		Boys (*n* = 65)		All children (*n* = 128)		*P*	Girls (*n* = 63)		Boys (*n* = 65)		All children (*n* = 128)	
		Mean	SD	Mean	SD	Mean	SD		Median	SE	Median	SE	Median	SE
Vitamin A (retinol equivalent) (μg)	210^a^	347	133	357	144	352	138	NS	349	17	352	18	350	12
Retinol (μg)	NA	164	52	177	72	171	63	NS	167	7	172	9	167	6
Beta-carotene (μg)	NA	1099	576	1082	520	1090	546	NS	1037	73	928	65	1015	48
Vitamin D (μg)	7^a^	0.59	0.21	0.59	0.25	0.59	0.23	NS	0.58	0.03	0.58	0.03	0.58	0.02
Vitamin E (mg)	4.2^b^	2.42	0.76	2.20	0.77	2.31	0.77	NS	2.38	0.10	2.12	0.10	2.26	0.07
Vitamin B_1_ (mg)	0.35^a^	0.393	0.129	0.367	0.118	0.380	0.123	NS	0.382	0.016	0.362	0.015	0.374	0.011
Vitamin B_2_ (mg)	0.35^a^	0.568	0.186	0.574	0.200	0.571	0.193	NS	0.584	0.023	0.577	0.025	0.580	0.017
Vitamin B_6_ (mg)	0.35^a^	0.59	0.19	0.56	0.17	0.57	0.18	NS	0.60	0.02	0.57	0.02	0.57	0.02
Folate (μg)	112^a^	86.9	24.4	82.7	23.7	84.8	24.0	NS	86.1	3.1	83.7	2.9	85.9	2.1
Vitamin B_12_ (μg)	0.7^a^	0.92	0.34	0.95	0.39	0.94	0.37	NS	0.93	0.04	0.93	0.05	0.93	0.03
Niacin (mg)	4.2^a^	3.90	1.33	3.56	1.12	3.72	1.24	NS	3.57	0.17	3.55	0.14	3.56	0.11
Vitamin C (mg)	28^a^	29.5	9.2	27.4	7.9	28.5	8.6	NS	31.1	1.2	26.0	1.0	28.4	0.8

*NA* not available, *P* significance; NS, *P* > 0.05

^a^70 % EAR

^b^70 % AI

**Table 5:** Mineral intake in the studied children during their stay at preschool

Nutrient	Reference values	Girls (*n* = 63)		Boys (*n* = 65)		All children (*n* = 128)		*P*	Girls (*n* = 63)		Boys (*n* = 65)		All children (*n* = 128)	
		Mean	SD	Mean	SD	Mean	SD		Median	SE	Median	SE	Median	SE
Calcium (mg)	560^a^	191	75	199	68	195	72	NS	185	10	200	8	191	6
Phosphorus (mg)	287^a^	346	103	346	104	346	103	NS	352	13	358	13	355	9
Magnesium (mg)	77^a^	82	23	79	20	80	22	NS	81	3	80	3	81	2
Sodium (mg)	700^b^	1240	371	1207	346	1223	357	NS	1221	47	1217	43	1220	32
Potassium (mg)	2170^b^	978	292	917	249	947	272	NS	938	37	940	31	939	24
Iron (mg)	2.8^a^	3.0	0.8	2.9	0.8	3.0	0.8	NS	3.0	0.1	2.9	0.1	3.0	0.1
Zinc (mg)	2.8^a^	2.8	0.8	2.8	0.8	2.8	0.8	NS	2.9	0.1	2.8	0.1	2.9	0.1
Copper (mg)	0.21^a^	0.31	0.09	0.30	0.08	0.31	0.09	NS	0.31	0.01	0.30	0.01	0.30	0.01
Manganese (mg)	1.05^b^	1.55	0.45	1.49	0.38	1.52	0.42	NS	1.50	0.06	1.38	0.05	1.47	0.04
Iodine (μg)	45.5^a^	51.5	18.3	50.2	16.9	50.9	17.5	NS	51.5	2.3	48.8	2.1	49.5	1.5

*NA* not available, *P* significance; NS, *P* > 0.05

^a^70 % EAR

^b^70 % AI

**Table 6:** The percentages of the studied children in the reference ranges for vitamin intake during their stay at preschool

Nutrient	Girls (*n* = 63)	Boys (*n* = 65)	All children (*n* = 128)	*P*
	%	%	%	
Vitamin A (retinol equivalent)				
Below 70 % EAR	11.1	13.8	12.5	NS
Retinol				
Above 70 % UL	0.0	0.0	0.0	#
Vitamin D				
Below 70 % EAR	100.0	100.0	100.0	#
Vitamin E				
Below 70 % AI	100.0	96.9	98.4	NS
Above 70 % UL	0.0	0.0	0.0	#
Vitamin B_1_				
Below 70 % EAR	36.5	41.5	39.1	NS
Vitamin B_2_				
Below 70 % EAR	11.1	15.4	13.3	NS
Vitamin B_6_				
Below 70 % EAR	6.3	10.8	8.6	NS
Above 70 % UL	0.0	0.0	0.0	#
Folate				
Below 70 % EAR	84.1	92.3	88.3	NS
Above 70 % UL	0.0	0.0	0.0	#
Vitamin B_12_				
Below 70 % EAR	23.8	24.6	24.2	NS
Niacin				
Below 70 % EAR	65.1	78.5	71.9	NS
Above 70 % UL	0.0	0.0	0.0	#
Vitamin C				
Below 70 % EAR	38.1	56.9	47.7	0.033
Above 70 % UL	0.0	0.0	0.0	#

*P* significance; NS, *P* > 0.05

#*P* cannot be calculated when percentage is 0.0 or 100.0

**Table 7:** The percentages of the studied children in the reference ranges for mineral intake during their stay at preschool

Nutrient	Girls (*n* = 63)	Boys (*n* = 65)	All children (*n* = 128)	*P*
	%	%	%	
Calcium				
Below 70 % EAR	100.0	100.0	100.0	#
Phosphorus				
Below 70 % EAR	22.2	27.7	25.0	NS
Above 70 % UL	0.0	0.0	0.0	#
Magnesium				
Below 70 % EAR	39.7	46.2	43.0	NS
Sodium				
Below 70 % AI	7.9	7.7	7.8	NS
Above 70 % UL	73.0	69.2	71.1	NS
Potassium				
Below 70 % AI	100.0	100.0	100.0	#
Iron				
Below 70 % EAR	38.1	43.1	40.6	NS
Above 70 % UL	0.0	0.0	0.0	#
Zinc				
Below 70 % EAR	44.4	52.3	48.4	NS
Above 70 % UL	0.0	0.0	0.0	#
Copper				
Below 70 % EAR	11.1	10.8	10.9	NS
Above 70 % UL	0.0	0.0	0.0	#
Manganese				
Below 70 % AI	9.5	6.2	7.8	NS
Above 70 % UL	11.1	4.6	7.8	NS
Iodine				
Below 70 % EAR	44.4	41.5	43.0	NS
Above 70 % UL	0.0	0.0	0.0	#

*P* significance; NS, *P* > 0.05

#*P* cannot be calculated when percentage is 0.0 or 100.0

## Discussion

The advantage of our study was that the probability that the preschool staff underreported children’s food intake is very low because the staff showed full involvement in recording children’s food intake and considered this difficult task as a challenge they should rise to. Energy intake in the studied preschoolers increased through the percentile categories for BMI which confirms that the preschool staff recorded the children’s food intakes very precisely. In the studies which aimed to assess daily food intake, energy intake was usually lower in obese subjects due to underreporting; however, underreporting in preschoolers has been little explored as discussed in the previous article [35]. In the previous study on 6-year-old Polish children, daily energy intake increased through all of the percentile categories, except for obese children whose energy intake was lower not only than in overweight children but even than in their peers with healthy weight [35]. Another study on Polish preschoolers reported that daily energy intake in children with tendency to overweight and in overweight children was lower even than daily energy intake of their underweight peers [53]. Also other studies on children of various age reported lower energy intakes in those with higher body weight [54–56].

Our study showed adequate intakes of total protein, monounsaturated fatty acids and cholesterol. There was also little risk of inadequate intakes of vitamin A, B_2_, B_6_, copper and manganese. However, although mean energy intake from available carbohydrates was within the recommended in most of the studied children, a substantial percentage of them fell below the recommendations. What is even more disconcerting, a high percentage of energy came from sucrose. Energy intake from sucrose was twice as high as the recommended intake of added sugars [38]; therefore, intake of all added sugars must have been even higher. Such high sucrose intake increases the risk of dental caries [57] and may adversely influence lipid profile [58] favouring atherogenesis.

It is unfavourable that most of the studied preschoolers were characterised by excessive intake of energy from total fat. Also, the structure of fatty acid intake was adverse due to high intake of saturated fatty acids along with very low intake of polyunsaturated fatty acids. High energy intake from saturated fatty acids increases the risk of developing atherosclerosis. Lowering intake of energy from this macronutrient is of greatest importance since ischaemic heart disease and stroke, which result from atherosclerosis, are the two most common causes of death all over Europe [59]. It is also emphasised that preventing atherosclerosis should start as early as in childhood [2]. On the other hand, energy intake from polyunsaturated fatty acids in the studied children during their stay at preschool was much lower than the recommended. Such low intake of energy from this macronutrient not only increases cardiovascular risk [60] but may also impair cognitive development [61].

Another adverse characteristic of the studied children’s dietary intake at preschool was intake of dietary fibre lower than 70 % of AI in all of the studied children. An intervention study in 7–11-year-olds showed that children accepted high-fibre snacks [62]; thus, introducing high-fibre foods to preschool menu should also be successful leading to increased dietary fibre intake. High fibre intake is essential in the prevention of diet-related diseases, such as obesity, type 2 diabetes and cardiovascular diseases [63], and decreases the likelihood of constipation [64].

Similarly to dietary fibre intake, also mean total water intake did not reach 70 % of AI and all of the studied children fell below 70 % of AI. It is noteworthy, that at preschool the children were not only served beverages with meals, but also had access to water so that they could drink it whenever they wanted to. However, children are often so absorbed in playing that they do not pay attention to being thirsty. That is why they should be encouraged by the preschool teachers to drink water. Although the children were asked to inform the teacher every time they wanted to drink water and the teachers did their best to control water drinking by the children, there is still a possibility that they may have failed to fully control it. Nevertheless, these results are not surprising since many studies reported inadequate water intake in children of various ages all over the world (e.g. [65, 66]). The preschool staff should be educated about the strategies of increasing water intake in children at preschool and about the importance of good hydration, including influence on physical and cognitive performance, reduced incidence of constipation, as well as possible association with diet-related diseases such as hypertension, fatal coronary heart disease or stroke [67].

Many inadequacies were found for vitamin intakes. Although mean intakes of vitamin B_1_, B_12_ and C were higher than 70 % of EAR, substantial percentages of children fell below 70 % of EAR, especially in the case of vitamin C. Major concerns are inadequate intakes of vitamin D, folate and niacin, and the fact that almost all of the studied children had intakes of vitamin E lower than 70 % of AI. Such inadequacies in children’s preschool diets increase the risk of diet-related diseases. Inadequate intake of vitamin D is not only linked to osteoporosis risk [68] but also cardiovascular diseases [69], type 2 diabetes [70] and most probably even cancer [71]. Folate and vitamin B_12_ were reported as factors protecting against coronary heart disease (e.g. [72]) and have been recognised to play an important role in bone health [73]. High folate intake also appears to reduce the risk of colon and breast cancer [74]. Additionally, inadequate intake of folate may be associated with mental degenerative disorders such as Alzheimer’s disease [75, 76]. Vitamin E seems to play a role in coronary heart disease prevention [74], especially together with vitamin C [74, 77]. Moreover, diets rich in vitamin C are associated with decreased risk of cancer [74]. Also niacin is important because it was reported one of the lipid-altering agents which decreases mortality due to heart attacks [78]. Therefore, it is indispensable to modify preschool diet to provide adequate amounts of all the vitamins to the studied children.

Also in the case of minerals, many inadequacies were found. Calcium intake indicates high risk of inadequate intake. It is surprising that preschool staff failed to provide adequate calcium content in the diet or failed to encourage children to eat more milk and dairy products. The importance of adequate calcium intake with milk has been widely spread in Poland for many years, even in special television campaigns. Such low calcium intake may predispose the studied preschoolers to osteoporosis later in life [79], since increased intake of dietary calcium/dairy products increases total body and lumbar spine bone mineral content [80]. It is even more disconcerting when taking into account the abovementioned inadequate intake of vitamin D. Another major concern is that mean intake of potassium was more than twice lower than 70 % of AI and that all of the studied preschoolers had lower intakes of this mineral than 70 % of AI. Adequate intake of potassium is one of the key factors in nutritional prevention of hypertension [81], thus the observed low intake of this mineral poses increased risk of developing future hypertension in the studied children. This effect may be aggravated by the observed excessive sodium intake which is another factor for hypertension development [81]. Adequate potassium and sodium intakes may also be important in osteoporosis prevention, since adequate potassium intake exerts protective effect on age-related bone loss [82], while high sodium intake increases the loss of urinary calcium [83].

Although mean intakes of magnesium, iron and iodine were higher than 70 % of EAR, and mean intake of zinc reached 70 % of EAR, more than 40 % of the studied preschoolers had intakes lower than 70 % of EAR. Therefore, content of these minerals in the preschool diet should be increased. It is very important since magnesium intake has been negatively associated with insulin resistance, type 2 diabetes, metabolic syndrome, hypertension, and cardiovascular diseases [84] and positively associated with bone mass [84]. Apart from the well-known effect of inadequate iron intake, which is anaemia, iron intake was also found to be inversely associated with coronary heart disease incidence [85]. Moreover, iodine, iron and zinc are suggested to be essential nutrients in cognitive performance and development of children [61], and preventing deficiencies of these nutrients is key in achieving full potential cognitive capacity and unnecessary loss of mental capacity [86].

The numerous inadequacies in children’s diets at preschool are the risk factors for developing diet-related diseases. Of course, preschool diet is not the whole day diet; therefore, the question arises whether foods eaten by the children outside preschool are capable of providing energy and nutrients in amounts and proportions which will balance the daily diet.

Our study also showed that dietary intakes at preschool were similar in the studied girls and boys, and only two statistically significant differences were found. Quite opposite, the studies which reported daily dietary intakes in children often showed statistically significant differences according to gender [35, 50, 87–89]. The preschool menu is the same for all children, and the preschool staff encourages each child to eat all meals, irrespective of gender, while parents most probably hold stereotypical beliefs that preschool girls have different nutritional needs than boys and that some foods are more suitable for children depending on their gender. These beliefs may lead parents to offering different foods to their sons and daughters and to serve different portion sizes, for example to feed their sons larger portions of meat than their daughters. This is very interesting and should be investigated in the future studies.

In comparison to the previously studied dietary intake at preschool in Polish 4–6-year-old children from Szczecin [31], intakes of energy and all nutrients, except for sodium, were higher. Sodium intake in the studied children was twice higher compared to their previously studied peers [31]. The preschoolers from Szczecin [31] were characterised by inadequate intakes of vitamin E, calcium and potassium, similarly to the studied children.

It is interesting that although the studied preschoolers were older than the previously studied Swedish preschool children aged 3 to 5 years [33], they were characterised by lower intakes of energy and total protein, much lower energy intakes from protein and fat, and much higher energy intakes from carbohydrates and sucrose. Intakes of micronutrients cannot be compared because mineral and vitamin intakes were presented either as daily intakes or as nutrient densities expressed per MJ.

In comparison to 3–4-year-old children from New York City [32], the studied preschoolers were also characterised by lower intake of total protein; however, intake of energy was higher. Moreover, intakes of vitamin A and E in the studied children were twice higher than those in children from New York City [32], intakes of magnesium and sodium were much higher, intakes of folate, vitamin B_12_ and iron were lower, intakes of vitamin D, niacin, vitamin C and calcium were much lower, while intakes of vitamin B_1_, B_2_, B_6_ and zinc were similar.

Previous studies which aimed to assess nutritional value of menus planned for children at preschools [4–9, 11, 13–18, 20–25] included various nutrients; however, all seven studies which included vitamin D [7, 11, 15, 20, 22, 24, 25] and eight [5–7, 11, 17, 18, 22, 24] out of ten [5–7, 11, 17, 18, 22–25] studies which included calcium reported their inadequate contents, whereas six [11, 17, 18, 23–25] out of seven [7, 11, 17, 18, 23–25] studies which included sodium and the only two studies [13, 16] which included sucrose reported their excessive contents. Moreover, the only four studies [4, 13, 16, 18] which included polyunsaturated fatty acids reported their inadequate content and all six studies [4, 13, 16, 21, 23, 25] which included saturated fatty acids reported their excessive content. Also a study designed to determine fat and fatty acid content in preschool meals in Wrocław, Poland [90], using chemical analysis showed excessive content of saturated fatty acids along with inadequate content of polyunsaturated fatty acids. In all of the abovementioned studies which aimed to assess nutritional value of preschools menus, the children’s actual dietary intake was not investigated. However, if the nutritional content of preschool menus was not balanced, it is not surprising that the result is preschoolers’ inadequate intake. The inadequacies found in the studied children’s dietary intake at preschool are in accordance with most of the inadequacies found in preschool menus in the previous studies [4–7, 11, 13, 15–18, 20–25, 90].

To prevent health effects resulting from inadequate and excessive intakes of nutrients, it is indispensable to plan balanced preschool menus. For this purpose, a dietician should be employed in preschools. In Poland, it is not a common practice.

## Conclusions

In conclusion, preschool diets need urgent improvement to prevent diet-related diseases in the studied preschoolers in the future. The inadequacies observed in these diets are in accordance with the previously reported inadequacies in menus planned for preschoolers. More research is needed to investigate dietary intake of children during their stay at preschool. Common regulations worked out for all preschools in the European Union would be a good way to provide adequate nutrition to preschool children. One of such regulations which should be imposed by the European Union on preschools should be the obligation to employ a dietician.
